# Structure–Stability Relationships in Pt-Alloy Nanoparticles Using Identical-Location Four-Dimensional Scanning Transmission Electron Microscopy and Unsupervised Machine Learning

**DOI:** 10.1021/acsnano.4c12528

**Published:** 2025-01-07

**Authors:** Ana Rebeka Kamšek, Francisco Ruiz-Zepeda, Marjan Bele, Anja Logar, Goran Dražić, Nejc Hodnik

**Affiliations:** † Department of Materials Chemistry, National Institute of Chemistry, Hajdrihova 19, 1000 Ljubljana, Slovenia; ‡ Faculty of Chemistry and Chemical Technology, University of Ljubljana, Večna pot 113, 1000 Ljubljana, Slovenia; § University of Nova Gorica, Vipavska 13, 5000 Nova Gorica, Slovenia

**Keywords:** 4D-STEM, electrocatalysis, IL-TEM, alloy ordering, unsupervised algorithms, structure−stability relationship, platinum alloy

## Abstract

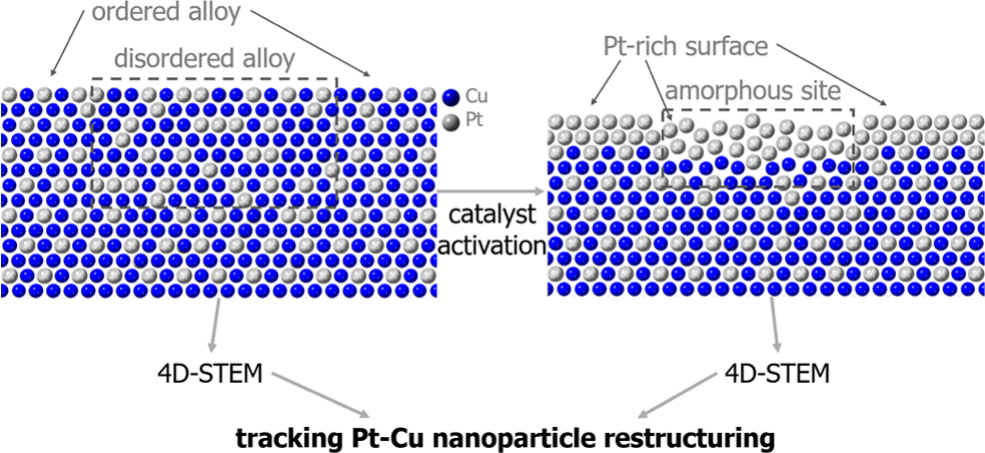

Nanoparticulate electrocatalysts for the oxygen reduction reaction are structurally diverse materials. Scanning transmission electron microscopy (STEM) has long been the go-to tool to obtain high-quality information about their nanoscale structure. More recently, its four-dimensional modality has emerged as a tool for a comprehensive crystal structure analysis using large data sets of diffraction patterns. In this study, we track the alternations of the crystal structure of individual carbon-supported PtCu_3_ nanoparticles before and after fuel cell-relevant activation treatment, consisting of a mild acid-washing protocol and potential cycling, essential for forming an active catalyst. To take full advantage of the rich, identical location 4D-STEM capabilities, unsupervised algorithms were used for the complex data analysis, starting with *k*-means clustering followed by non-negative matrix factorization, to find commonly occurring signals within specific nanoparticle data. The study revealed domains with (partially) ordered alloy structures, twin boundaries, and local amorphization. After activation, specific nanoparticle surface sites exhibited a loss of crystallinity which can be correlated to the simultaneous local scarcity of the ordered alloy phase, confirming the enhanced stability of the ordered alloy during potential cycling activation conditions. With the capabilities of our in-house developed identical-location 4D-STEM approach to track changes in individual nanoparticles, combined with advanced data analysis, we determine how activation treatment affects the electrocatalysts’ local crystal structure. Such an approach provides considerably richer insights and is much more sensitive to minor changes than traditional STEM imaging. This workflow requires little manual input, has a reasonable computational complexity, and is transferrable to other functional nanomaterials.

Fuel cell electrocatalysts stand at the forefront of commercializing hydrogen as a viable alternative to fossil fuels in the transport and stationary power generation sectors. Those functional nanomaterials currently represent a bottleneck to fuel cell commercialization due to their high price, as they are commonly based on noble metals. Electrocatalysts for the oxygen reduction reaction (ORR) inside a proton exchange membrane fuel cell specifically use scarce platinum. Today, they are commonly made of platinum alloy nanoparticles, which contain abundant transition metals such as Cu, Co, Ni, or Fe. Alloyed nanoparticles are dispersed over high-surface-area support like carbon, drastically improving platinum utilization while retaining good catalytic properties.^1−3^ Despite their successful development, further fundamental investigation into their structure–activity and structure–stability relationships is essential to reach their maximum potential.

Structural features govern the catalytic properties of alloyed nanoparticles. One example is a better ORR performance in both activity and stability tests for nanoparticles, encapsulated by a Pt-rich surface.^1^ Several strategies were reported to create the overlayer^4^ and tune its thickness.^5^ Furthermore, the synthesis of intermetallic structures resulted in more active and stable electrocatalysts.^1,6−8^ Alloy ordering is thought to improve the catalytic properties due to the enhanced stability of the less noble metal.^9^*In situ* studies correlated a higher degree of order with better ORR activity and durability^10^ and demonstrated the separation of the alloying and ordering stages.^11^ However, only a handful of references also consider the physical placement of ordered domains inside nanoparticles, for example using atomically resolved imaging to show an ordered shell and a disordered core in a Pt–Cu nanoparticle,^12^ tracking alloying and ordering in Pt–Fe nanoparticles,^13^ and specifying chemical order at the atomic scale for a Pt–Fe nanoparticle.^14^ Such complexity inevitably results in an exclusive atomic arrangement and thus structure of each nanoparticle.^15^ Therefore, a bottom-up approach is needed to study their structure–function relationships.^3^

Changes to the electrocatalyst structure during operation occur at the nanoscale. Scanning transmission electron microscopy (STEM) is a versatile tool that can acquire that information down to the atomic scale and is thus indispensable when characterizing nanomaterials. Identical-location STEM (IL-STEM), where an identical site or particle is characterized consecutively, is especially useful when investigating local changes before and after a certain *ex situ* change-inducing protocol, including studying electrochemical aging in nanocatalysts.^16,17^ It proved itself useful many times over in the field of ORR electrocatalysis and continues to offer information that is more objective and reliable than *ex situ* imaging of randomly picked locations.^3,18,19^ Even though it does not provide *in situ* data, comparing the starting and final configurations of a specific site can explain the possible mechanism behind the transformation. While it is true that *in situ* imaging using an electrochemical cell would provide real-time insights, it would most likely mean sacrificing atomic-scale information. Modern imaging modalities promise an even better utilization of identical-location imaging in the context of functional materials.

Four-dimensional STEM (4D-STEM) is a state-of-the-art method that collects diffraction patterns with a pixelated detector while scanning a thin sample with an electron beam. This creates massive data sets of tens of thousands of patterns with comprehensive information about the local crystal structure. The electron beam should in principle be close to a zone axis of the investigated structure to achieve an adequate diffraction contrast,^20^ but 4D-STEM nonetheless reduces the need for atomic resolution imaging compared to conventional STEM since the diffraction patterns retain crystal structure information at any magnification. The technique also reduces the impact of sample drift and other distortions on a crystal structure analysis since individual patterns are recorded at once. Lastly, 4D-STEM offers more data compared to conventional STEM and does not reduce entire spatial distributions of the scattered electrons to scalar numbers.

4D-STEM has already been successfully applied to several crystal structure studies at the nanoscale.^21−23^ Analyzing tens of thousands of diffraction patterns by hand is out of the question due to the sheer amount of data. Automating the analysis not only speeds it up and ensures its objectivity but also offers information that would be impossible to obtain manually.^24^ There have already been numerous studies where the analysis of STEM images was automated^25^ as well as software solutions for 4D-STEM.^26,27^ Some of those are dedicated to orientation mapping for crystalline materials,^28−30^ generally aimed at systems where all phases were already identified.

Unsupervised machine learning, on the other hand, offers outstanding possibilities for analyzing large amounts of entirely unlabeled data which is handled without prior knowledge about the sample or data acquisition method. Among such algorithms, clustering groups data points into discrete groups or clusters. There have already been several successful attempts in 4D-STEM to cluster the data into physically relevant groups, for example as an exploratory data analysis approach,^31,32^ to reveal lattice deformations,^33^ stacking order in multilayer nanomaterials,^34^ and to segment twinned crystallites.^35^

Clustering, however, usually fails to consider the possibility of one data point including several different signals, which can very well be the case when imaging high surface area nanoparticulate electrocatalysts. Significant structure overlap can occur due to a large number of nanoparticles that are generally rotated randomly and exhibit a variety of crystal structures and defects, which is why other algorithms need to be considered. Dimensionality reduction can reduce a high-dimensional data set to a low number of eigenvectors, and can therefore determine significant information within it. Examples of studies using dimensionality reduction on 4D-STEM data include general data exploring,^32,36^ denoising,^37^ confirming strain as a dominant feature,^38^ and for crystallite segmentation and analysis.^35,39−46^ So far, such approaches have not been widely explored in electrocatalysis.

In this study, we demonstrate an identical-location 4D-STEM approach on an ORR electrocatalyst with carbon-supported Pt–Cu nanoparticles that underwent acid washing and potential cycling activation. We analyzed the 4D-STEM data sets using clustering and dimensionality reduction to obtain objective information about the local crystal structure. Identical location data enabled us to establish a link between the local crystal structure and the onset of degradation with observable local collapse of the initial crystal structure. Coupled with simulated 4D-STEM data, X-ray diffraction (XRD), scanning electron microscopy (SEM), energy-dispersive X-ray spectrometry (EDX), and electron energy loss spectroscopy (EELS), this is a thorough study of the local structure–stability relationship of individual Pt–Cu nanoparticles.

## Results and Discussion

The present study encompasses a detailed investigation of the structure–stability relationship of an ORR electrocatalyst consisting of carbon-supported PtCu_3_ nanoparticles. After an initial structural characterization of the sample, an identical-location 4D-STEM study was carried out on individual nanoparticles to study surface degradation mechanisms during sample treatment. Using advanced characterization methods and data analysis algorithms, this study builds on existing materials science knowledge to deliver a deeper understanding thanks to data-driven approaches.

### *Ex Situ* Characterization of the As-Synthesized Sample

**Figure 1:**
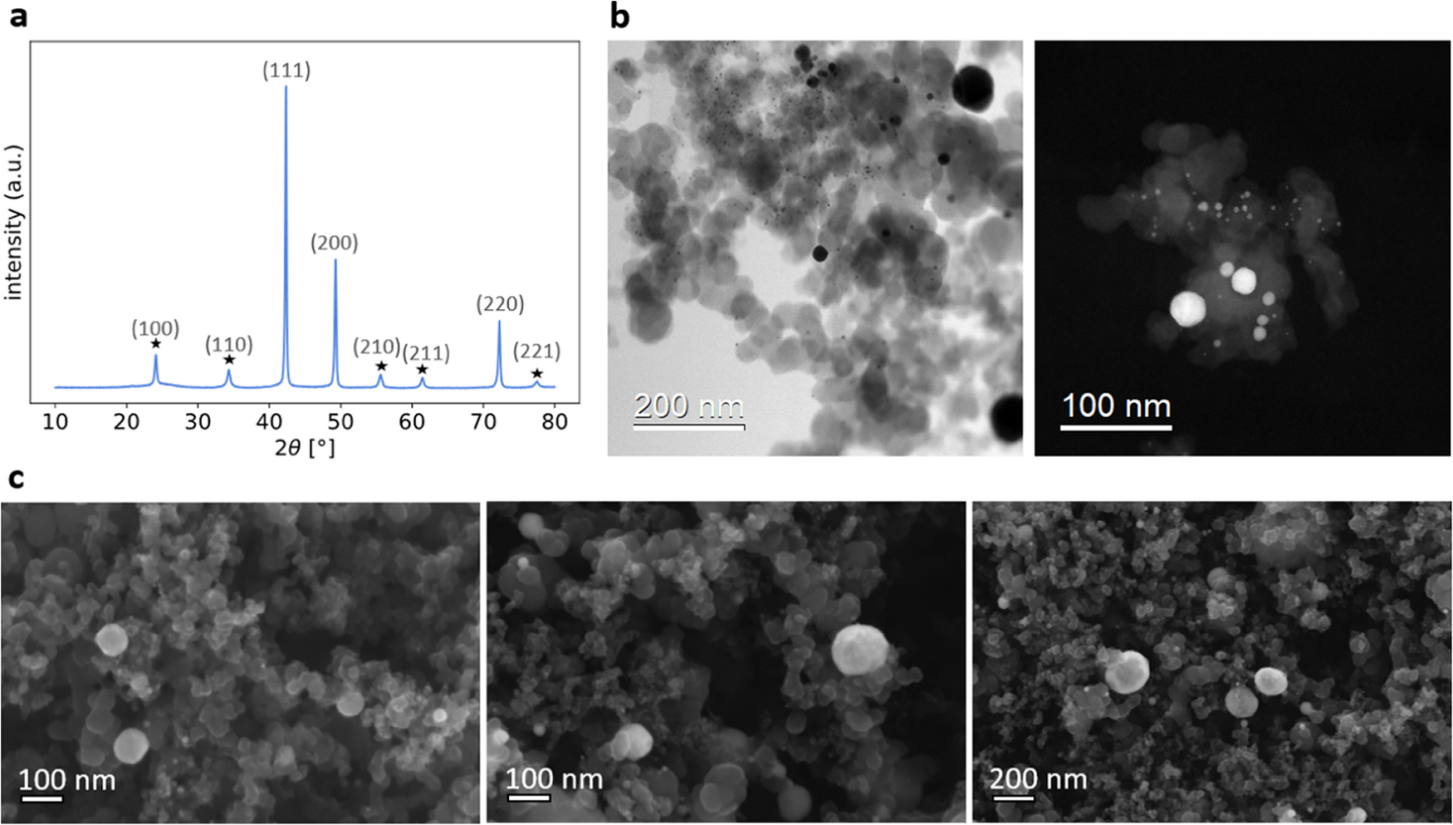
(a) XRD pattern of the investigated PtCu_3_/C sample. Star markers denote superstructure diffraction maxima, characteristic of the ordered alloy crystal phase. (b) BF- and HAADF-STEM images of the sample showing the carbon support morphology and the Pt–Cu nanoparticles. (c) SEM images of the sample.

A powder catalyst consisting of carbon-supported PtCu_3_ nanoparticles was synthesized using an in-house procedure. Figure 1a presents an X-ray diffraction pattern, where the most prominent three diffraction maxima corresponding to (111), (200), and (220) planes are characteristic of the disordered Pt–Cu alloy (*Fm*3̅*m*) phase, and the rest of the maxima reveal the presence of the ordered PtCu_3_ alloy (*Pm*3̅*m*) phase. The intensities of the diffraction maxima confirm a mixture of both alloy crystal phases while the broad signal at ∼25° belongs to the partially graphitic carbon support.

Figure 1b features bright-field (BF) and high-angle annular dark-field (HAADF) STEM images of the PtCu_3_/C electrocatalyst. In the bright-field image, the morphology of the carbon is visible. Carbon particles, spanning a few tens of nanometers in diameter, form aggregates that provide a high surface area for dispersing the catalytic nanoparticles. In both images, we can observe Pt–Cu nanoparticles from 4 to 100 nm in diameter. Figure 1c includes SEM images to show further the carbon morphology and Pt–Cu nanoparticles’ faceted shape. More images can be found in Figure S1. We note that the wide particle size distribution is beneficial for our study, as it allows us to select particles of specific sizes for detailed analysis. It is not intended that this material represents an optimized performing electrocatalyst.

**Figure 2:**
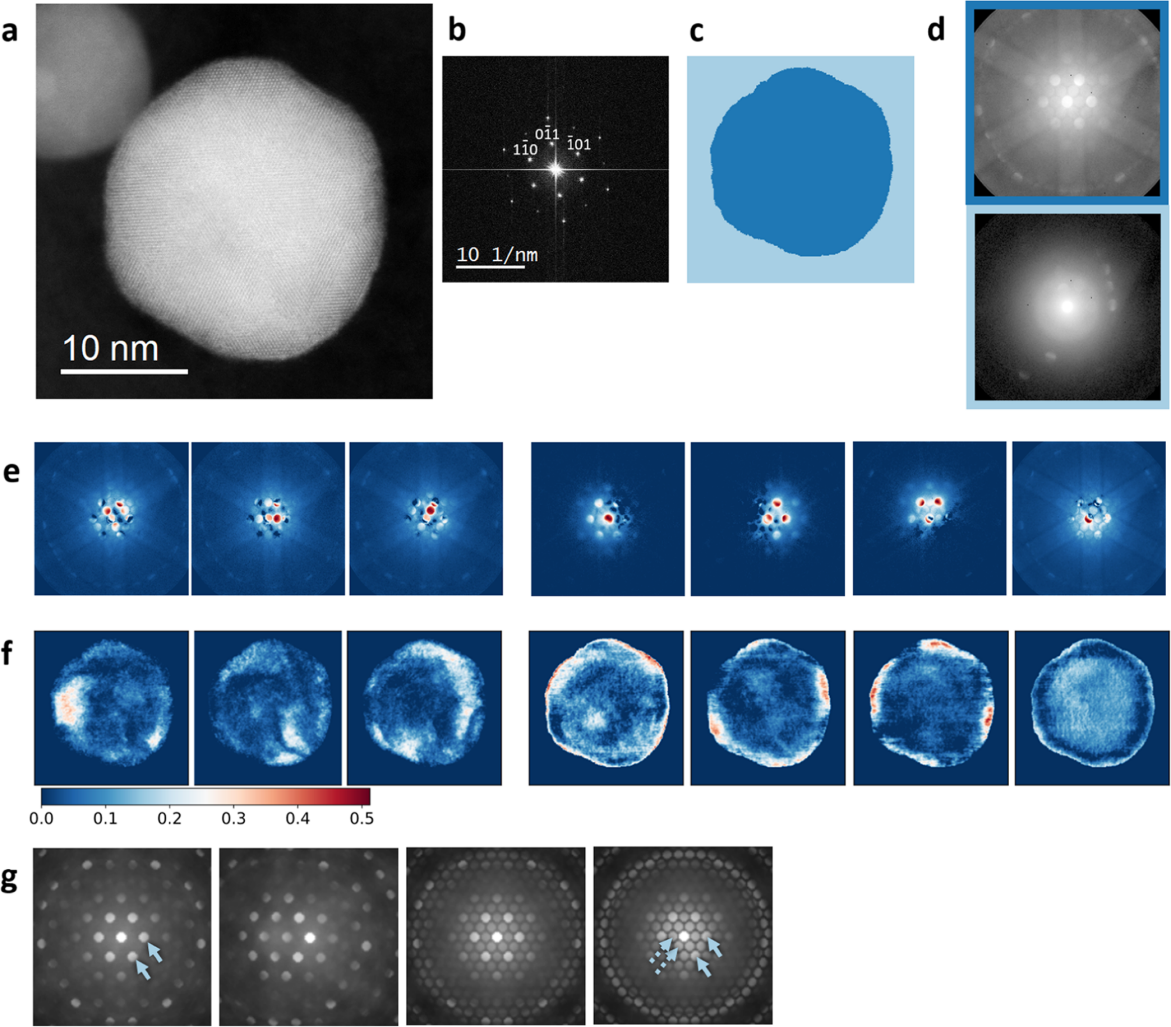
(a) HAADF-STEM image of a Pt–Cu nanoparticle in the [111] zone axis. (b) FFT of the HAADF-STEM image. (c, d) Clustering with color-coded labels (c) and the cluster average diffraction patterns with a border of the same color as their label (d). (e, f) The representative diffraction patterns, determined with NMF (e), and their loading maps (f). The color scale corresponds to the extent to which each calculated pattern is present in the overall diffraction signal. Square roots of diffraction pattern intensity values are plotted. (g) Simulated diffraction patterns of relevant Pt–Cu alloy phases in the [111] zone axis. From left to right: the disordered alloy (arrows denote characteristic Bragg disks), the disordered alloy with a 3° tilt away from the zone axis, the 50:50 mixture of the ordered and disordered alloys, and the pure ordered alloy (dashed arrows denote superstructure Bragg disks while full arrows denote disks that are also present in the disordered alloy).

Selected nanoparticles underwent a detailed 4D-STEM analysis. Figure 2a includes a HAADF-STEM image of one such particle with its fast Fourier transform (FFT) in Figure 2b. It was imaged in a [111] zone axis, which can be inferred from the FFT and directly from the image. Figure S2 includes its BF-STEM image, average diffraction pattern, and reconstructed images from the 4D-STEM data set.

Since the data set naturally included the nanoparticle surroundings, i.e., the carbon support and a smaller, out-of-focus neighboring nanoparticle, it was necessary to isolate the diffraction patterns, belonging strictly to the nanoparticle under investigation. To do so in an automated manner, we turned to unsupervised learning. *K*-means clustering was chosen thanks to its successful results, acceptable computational complexity, and simplicity of use. The algorithm requires the user to provide the desired number of clusters. In this case, two clusters were sufficient and yielded a result where the entirety of the studied nanoparticle was included in a single cluster.

Figure 2c shows the *k*-means clustering results using two clusters, performed on the 4D-STEM data. Colors are used to illustrate the cluster labels in real space and the average diffraction pattern of each is depicted in Figure 2d. One cluster represents the Pt–Cu nanoparticle while the other includes the rest of the imaged area. The algorithm segmented the data set meaningfully despite having no prior knowledge of the data or the imaging method. The [111] zone axis, used in this case, results in a characteristic 6-fold symmetry, evident in the nanoparticle cluster diffraction pattern in Figure 2d. In contrast, the other cluster diffraction pattern is a mixture of a ring signal, coming from the carbon support, and several Bragg disks, coming from the other parts of the imaging area.

The diffraction patterns from the nanoparticle cluster were then used for dimensionality reduction. Non-negative matrix factorization (NMF) was performed to extract seven representative diffraction patterns, which was a reasonable value since no additional information appeared when increasing that number further. The calculated patterns are depicted in Figure 2e. Square roots of diffraction pattern intensities were plotted to better visualize the faint features, and the original NMF results can be found in Figure S5. The left-most three patterns include a signal, consistent with the ordered PtCu_3_ alloy phase, as evident from the superstructure Bragg disks closest to the central one. Here, it should be noted that each pattern does not necessarily mean a particular crystal structure, but rather a notable signal within the data set, which means that there can be more than one pattern, consistent with one crystal structure.

Several simulated diffraction patterns are depicted in Figure 2g to help understand the NMF results. Models, used for simulations, can be found in Figure S3. Indeed, the disordered and the ordered Pt–Cu alloys differ by the presence of superstructure Bragg disks. They also exhibit different Higher-Order Laue Zone (HOLZ) lines that form rings in the outermost part of the patterns but are not as prominent in individual experimental diffraction patterns. When imaging a mixture of the two phases, they share certain disks, and the disk intensities depend on the phase fractions. Thus, patterns simply featuring a signal in the place of superstructure disks do not necessarily depict a pure ordered alloy but should be understood as a possible mixture of phases.

The disordered alloy patterns could also be associated with a Pt-rich nanoparticle surface, as the Pt lattice has the same symmetry as the disordered Pt–Cu alloy and there are no significant differences among disk intensities using the chosen instrumental parameters. Additionally, disk intensities will change when a slight tilt away from the zone axis occurs, leaving us with fewer disks that are intense enough to be discerned from the noise. The pattern interpretation should therefore be careful and consider different crystal structures, mixtures, and tilts. Using NMF on 4D-STEM data was validated on a simulated data set as summarized in Figure S4. For comparison, we also performed principal component analysis (PCA) on the experimental data set from Figure 2 and included the results in Figure S6. PCA results were found to be less readily interpretable than results from NMF, similar to previous reports.^41^

Figure 2f shows loading maps, associated with each calculated diffraction pattern. Intensities represent the spatial abundance of each pattern, that is, what fraction of an individual experimental diffraction pattern at a specific site is associated with that calculated pattern. One or more corresponding domains can be discerned from the maps for each calculated pattern. In this case, we can attribute the left-most three maps to alloy ordering, as their corresponding diffraction patterns include signal, expected from an ordered PtCu_3_ alloy. The ordered domains are located in the outer part of the particle, forming an ordered alloy shell around a disordered alloy core of the nanoparticle, as reported previously for a similar system.^12^ Parts of the ordered domains are located at the particle surface and are thus in direct contact with particle surroundings.

More generally, this approach would enable us to highlight any domains that would exhibit characteristic diffraction—not only different space groups but also, for example, twin boundaries and crystallite orientations. The advantage of this approach is not needing to supply any prior knowledge about what we expect to see.

Nonetheless, the reliability of result interpretation highly depends on the present structures, the zone axis, and imaging parameters. In the [111] zone axis, identifying (partially) ordered crystallites was straightforward because the superstructure Bragg disks are visible at the imaging parameters used in this study. The mere phase identification is less straightforward in certain other cases. Figure S7 includes the results for a twinned Pt–Cu nanoparticle in the [110] zone axis.

Additionally, some parts of the nanoparticle can be highlighted in multiple maps. This already proves the need to consider structure overlap when imaging nanoparticulate electrocatalysts which is not possible with *k*-means clustering that assigns each data point to exactly one cluster. Distinguishing this is more effective with dimensionality reduction, as the overlap between crystal phases no longer poses a problem. This is an advantage of using 4D-STEM over conventional atomically resolved STEM, which provides individual 2D projections of the structure under investigation.

### Identical-Location 4D-STEM

One 4D-STEM snapshot of the investigated nanoparticle provided us with information about the physical placement of the ordered alloy phase. Identical-location imaging takes us a step further and enables a direct comparison of a chosen site or a nanoparticle before and after induced changes.^16^ In this study, we carried out two steps to alter the sample, acid washing and potential cycling activation.

Acid washing is a chemical activation method for Pt-alloy-based ORR electrocatalysts which removes the less noble metal from the outermost layers of nanoparticles to form a Pt-rich surface.^47−49^ This is important to activate the surface and prevent contamination of the whole proton exchange membrane fuel cell system with leached metal cations. The chosen protocol is a milder version of an activation protocol compared to industry-relevant routines, as we intended to induce minimal changes to check the robustness of the methodology against minor alternations between data sets. Figure S8 contains an IL-HAADF-STEM image of the nanoparticle after acid washing, a comparison to the as-synthesized state, and 4D-STEM analysis results. Indeed, after close inspection, one can recognize that the nanoparticle exhibited only minor changes. The results can be directly connected to the first set and only reveal slight particle reshaping.

EDX results in the form of maps and line scans, summarized in Figure S9, now more clearly reveal a minor enrichment of the surface with platinum (more red color on the edge of the nanoparticle), signifying copper dissolution from the outermost atomic layers as expected for acid washing. These results show that only combining several methods returns comprehensive information that addresses both chemical composition changes and crystal structure information.^50^

The second sample treatment step was potential cycling activation, performed on the TEM grid in a modified floating electrode (MFE) setup. This method is based on a three-electrode setup, where the TEM grid with the deposited sample assumes the role of the working electrode. This makes MFE a convenient way to induce changes to the sample electrochemically and enables identical-location imaging of electrocatalysts at different scales.^51^

**Figure 3:**
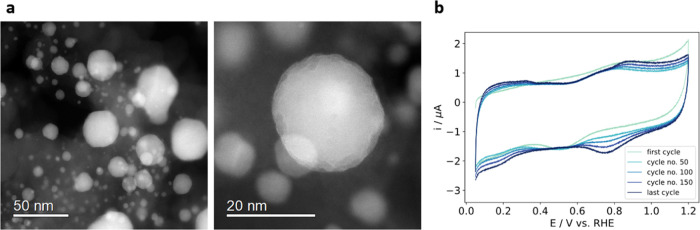
(a) HAADF-STEM images of the investigated PtCu_3_/C sample after potential cycling activation. (b) Cyclic voltammograms, recorded during potential cycling activation of the TEM grid with the PtCu_3_/C sample.

Figure 3a depicts STEM images of the activated sample. The close-up shot of one Pt–Cu nanoparticle reveals a rugged surface in contrast with the faceted shapes of nanoparticles after synthesis and agrees with previous literature reports.^18^ The cyclic voltammograms in Figure 3b exhibit relatively low electric currents due to a small amount of catalyst on the working electrode, but the signal is consistent with the electrochemical response of platinum in the chosen potential window under an inert atmosphere, especially in the last cycle where a Pt signature indicates a formation of a Pt-rich surface.^51^ A Pt-rich surface is confirmed with EDX results as shown in Figure S9. A modified surface and a larger electrochemically active surface area are a desired outcome of activation protocols for ORR electrocatalysts, which in turn makes catalyst conditioning a very important step in industrial settings.^47,48^

**Figure 4:**
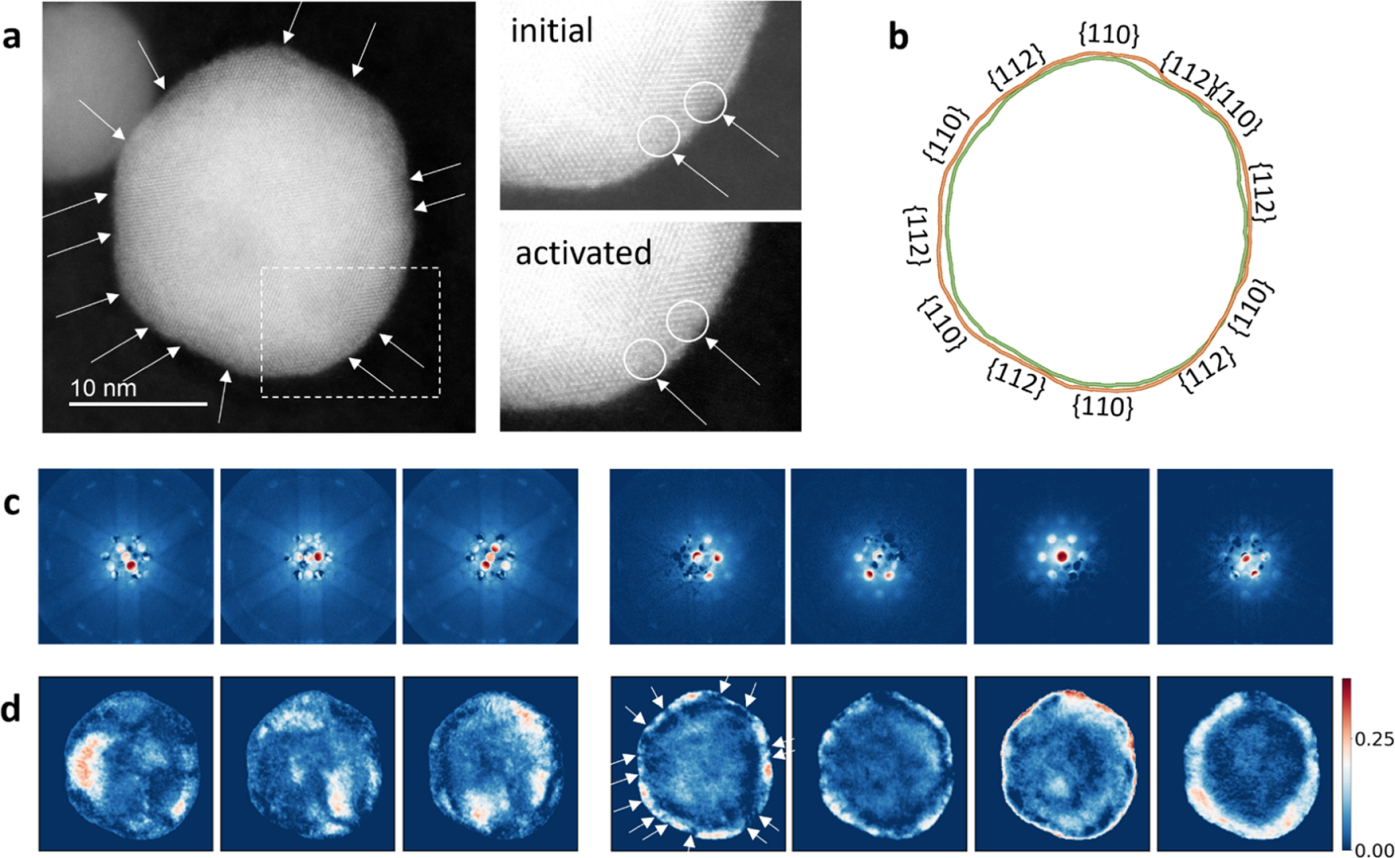
(a) HAADF-STEM image of a Pt–Cu nanoparticle after potential cycling activation. Arrows denote sites with local amorphization. The part within the dashed rectangle is visualized on the right with enhanced contrast and compared to the initial state. (b) Overlaid nanoparticle outlines before (red) and after (green) activation with Miller indices of crystal plane families. (c, d) The representative diffraction patterns, determined with NMF (c), and their loading maps (d). The color scale corresponds to the extent to which each calculated pattern is present in the overall diffraction signal. White arrows on one of the loading maps match the arrows on the HAADF-STEM image. Square roots of diffraction pattern intensity values are plotted.

Identical-location imaging was performed as previously. Figure 4a includes a HAADF-STEM image of the investigated Pt–Cu nanoparticle where sites with a collapsed crystal structure can be observed as nanometer-sized amorphous regions on the particle surface. When comparing parts of the nanoparticle surface to the initial state, it is apparent that the highlighted sites lost their crystallinity. Only the local short-range order is affected, while the rest of the nanoparticle retained its crystalline symmetry. In addition, a minor particle shrinkage is observed as shown in Figure 4b. The final diameter was approximately 3.6% smaller than the initial value.

While spotting local amorphization is possible by consulting a HAADF-STEM image, success is not guaranteed if the atomic resolution is compromised, and a manual approach is slow and subjective. These risks can be mitigated using 4D-STEM and automated data analysis. Figure 4c depicts NMF results for the 4D-STEM of the activated particle, where one of the loading maps in Figure 4d features signal gaps denoted with arrows that directly correspond to the local collapse of the crystal structure as observed with HAADF-STEM. Square roots of diffraction pattern intensities were plotted to better visualize the faint features, and the original NMF results can be found in Figure S5. The calculated diffraction pattern, used to construct that particular loading map, is consistent with a signal of a *Fm*3̅*m* crystal structure, shared both by a disordered Pt–Cu alloy and a Pt-rich surface. Therefore, it is not surprising that amorphous regions would not be highlighted there.

The signal gaps are, however, not equally as prominent in certain other loading maps. For example, the second-to-last map, looking from left to right in Figure 4d, does not feature any signal gaps on the nanoparticle surface. Its corresponding calculated diffraction pattern highlights the central Bragg disk most prominently, which is also the only noteworthy signal that can be expected from amorphous structures. The second-to-last loading map indeed highlights parts that mostly correspond to sites with local amorphization—although it is important to stress again that each map is obtained using the entirety of the signal in each calculated diffraction pattern which may feature a mixture of signals. Understanding the principles behind 4D-STEM and NMF is crucial to verify that all results corroborate the overarching story.

A previous study showed that a local surface enrichment with Cu resulted in sites being more susceptible to pore formation during electrochemical cycling.^18^ In our case, however, the EDX investigation of several Pt–Cu nanoparticles did not reveal significant inhomogeneities in the surface chemical composition after synthesis that could be connected to the observed nanometer-sized amorphous regions after activation. Besides Cu-rich sites, differences in the crystal structures could in principle also govern the local structure–stability relationship.

**Figure 5:**
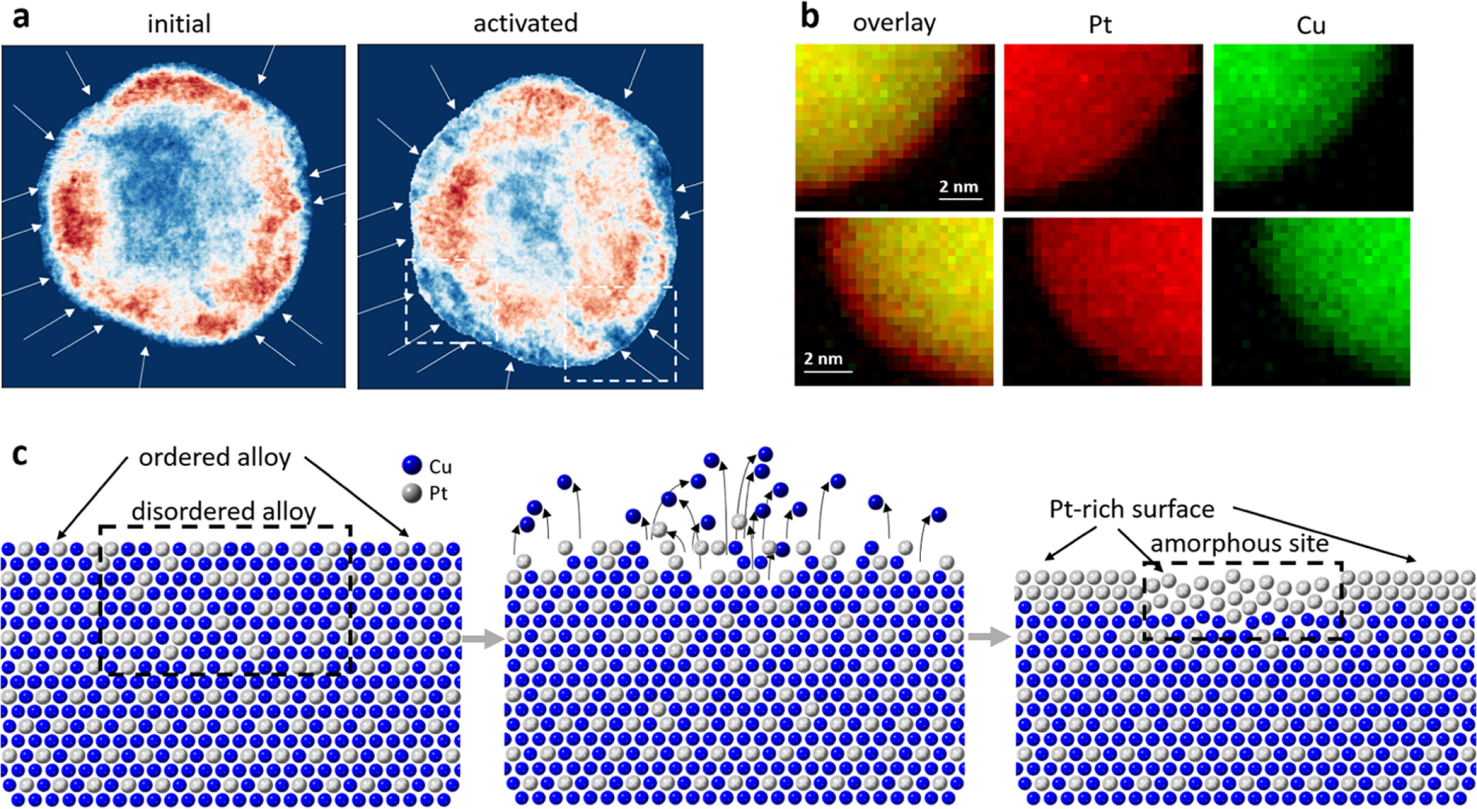
(a) Maps depicting alloy ordering within a Pt–Cu nanoparticle after synthesis (left) and after potential cycling activation (right). White arrows denote sites with observed local amorphization after activation. Each map is normalized to its respective maximum value. White dashed rectangles denote regions for EELS mapping. (b) EELS elemental maps for two parts of a nanoparticle after activation. Pt signal is in red and Cu is in green. (c) A schematic depiction of the proposed mechanism for the local amorphization on the Pt–Cu nanoparticle surface.

Figure 5a shows where an ordered alloy structure is present within the investigated Pt–Cu nanoparticle after synthesis and after potential cycling activation. Interestingly, ordered domains often stretch to the particle surface in the initial state. After activation, however, the ordered alloy signal at the surface often vanishes, consistent both with a Pt-rich surface formation and local amorphization.

Both ordered alloy maps come with marked amorphous sites after activation, as determined previously for the HAADF-STEM image. Those sites likely predominantly form due to the selective dissolution of Cu atoms from the near-surface regions, followed by a partial collapse and diffusion of the leftover Pt atoms toward fcc lattice sites. Because Pt atoms do not occupy the expected fcc lattice positions, the site becomes locally amorphous.

It is systematically observed that amorphous sites are more common in places with a locally lower degree of ordering (or close to them), and significantly less common in places with a higher degree of alloy ordering. Interestingly, amorphous sites appear to be placed at characteristic distances from one another, with the majority of the distances between them being approximately 3 to 5 nm (Figure 4a). It is worth noting that slight sample drift may occur due to a longer acquisition time of the 4D-STEM data set compared to the HAADF-STEM image. Transferring markers to denote amorphous sites therefore comes with an error margin of a few tenths of a nanometer. Nonetheless, it is still possible to compare the two data sets as the uncertainty in arrow placement is an order of magnitude lower than the distances between them.

Figure 5c schematically depicts the proposed mechanism of this phenomenon. While Cu atoms dissolve from a less stable region, namely the disordered phase, there is inherently also the formation of low-coordinated or dangling Pt atoms prone to relocate. These mobile Cu and Pt atoms mark the beginning of amorphous site formation. Under these conditions, Pt atoms, mobilized either by dissolution followed by redeposition or surface diffusion, are more likely to rearrange locally on the nanoparticle rather than detach completely and remain in the electrolyte. While Cu dissolves, the remaining Pt atoms form a Pt-rich surface, which we confirmed with EELS. Elemental maps for two parts of the investigated nanoparticle can be found in Figure 5b. The Pt-rich surface does not exhibit a homogeneous thickness, as evident also from the EDX maps of other activated nanoparticles in Figure S9.

It should be emphasized that the maps in Figure 5a are not normalized to the same value but to each respective maximum value. They are constructed using different sets of NMF eigenvectors and a direct comparison of the values in the figure is therefore pointless.

Besides the chemical composition and crystal structure, the local coordination number can also impact the surface site stability. Surface defects such as steps and edges can be expected to behave differently compared to sites in the middle of certain low-index surface facets since a different coordination number can change the local pH in the surrounding electrolyte. The impact of the surface coordination number on ORR activity and stability was investigated in detail in previous reports.^52^

In this study, however, the predominantly attacked sites seemed to be more connected to the crystal structure rather than to surface defects. The stability of the ordered and disordered Pt–Cu alloy structures was previously investigated theoretically by calculating the vacancy formation energy of individual Cu atoms. Cu stability was indeed determined to be higher in intermetallic structures which goes in line with the present experimental findings.^9^

Probabilities for surface changes likely follow a priority list: the local chemical composition has the largest effect, followed by alloy ordering and local coordination number. Less stable regions exhibit a higher probability of degradation events, and changes occur randomly when all regions are equally as stable. Dealloying, surface diffusion, and redeposition are processes that can occur simultaneously and are interrelated. Those nanocorrosion processes are an opportunity to form amorphous sites, and the resulting local collapse of crystallinity can then be observed and explained with 4D-STEM.

Even though identical-location imaging has its limitations, statistics being one of them, it represents the behavior of chosen sites throughout an entire experiment, which would be impossible with random location STEM imaging of an admittedly larger number of nanoparticles. This enables recognizing trends rather than a simple recognition of the present crystal structures, which makes the interpretation more telling, especially in the context of catalyst stability and conditioning. Identical location micrographs of individual nanoparticles offer more accurate conclusions regarding their restructuring. Using 4D-STEM for an identical location study offers an additional advantage as the evolution of the crystal structure can be tracked locally. Although the overlap between some ordered and disordered alloy disks and the presence of the background make phase quantification unfeasible, phase identification nonetheless remains possible in all collected data sets.

Identical-location 4D-STEM together with unsupervised algorithms is a powerful method for probing the local structure–stability relationship of nanocomposite electrocatalysts, and is an appropriate accompaniment to studies, investigating bulk catalysts. This method can be applied to other nanomaterials, where crystal phase mapping would provide meaningful information that could be connected to the material’s functional properties. Additionally, the data analysis pipeline is well-suited for automating complex and large-scale data set analysis such as in IL-4D-STEM, a task that would be practically impossible to carry out manually.

## Conclusions

In this study, we investigated the crystal structure of a Pt–Cu/C nanoparticulate ORR electrocatalyst using XRD, identical-location STEM, and IL-4D-STEM supported by unsupervised machine learning analysis consisting of *k*-means clustering and NMF. A mild acid-washing protocol and potential cycling activation were used to induce structural changes which were then tracked at identical locations. IL-HAADF-STEM revealed minor particle reshaping and local loss of short-range order (amorphization) at specific surface sites which correlated well with diffraction data, and a Pt-rich surface was confirmed with IL-EDX and EELS. Nanoparticle surface sites that exhibited local loss of crystal structure can be correlated to alloy ordering, as ordered domains are more stable under the conditions of potential cycling activation.

This work presents an important methodological step as 4D-STEM and corresponding data analysis are still under-utilized in electrocatalysis. The presented approach requires little manual input and is not limited to nanoparticulate electrocatalysts. 4D-STEM offers several advantages over using solely conventional STEM imaging. Since nanoparticles are 3D objects, the two-dimensional projections may include overlapping signals in the imaging direction. Data acquisition and analysis that considers that overlap is thus a welcome step forward compared to studies that disregard this aspect. In this case, we recognize that each experimental diffraction pattern can be understood as a sum of common signals, determined with unsupervised algorithms. Furthermore, unsupervised algorithms can also reveal unexpected domain differentiation or unknown features, unlike conventional deterministic approaches or supervised learning which requires labeled data. Last but not least, 4D-STEM offers crystal structure information even if real-space images do not offer atomic resolution as long as the imaged structure is close enough to a zone axis for diffraction patterns to feature relevant Bragg disks.

In the future, where computational complexity would be less of a concern, real-time exploratory data analysis might serve as a useful tool to the microscope operator during imaging and possibly help them collect more representative data rather than imaging arbitrarily chosen regions that may or may not hold comprehensive information on the structure under investigation. Achieving cooperation between humans and machines and among different characterization methods will enable even smarter design of functional materials, capable of solving humanity’s problems.

## Methods

### Pt–Cu/C Electrocatalyst Synthesis

A PtCu_3_/C sample was synthesized similarly to previous reports.^53^ In brief, a modified sol–gel method was used to mix the metal reactants at the molecular level. First, 0.08 g of hydroxyethyl cellulose (Merck, Germany) was dissolved in 6 mL of water by heating the mixture to 90 °C to ensure complete dissolution. After cooling the solution to 50 °C, 0.18 g of copper(II) acetate monohydrate (Honeywell, Germany) and 0.12 g of tetraamine platinum(II) nitrate (Sigma-Aldrich, Germany) were added and dissolved. To the resulting viscous solution, 0.25 g carbon black (Vulcan XC72R, Cabot) was added and then stirred to achieve a uniform dispersion. The mixture was then frozen with liquid nitrogen and freeze-dried to obtain a dry composite powder.

The freeze-dried powder was then heated to 250 °C with a heating rate of 2 °C/min in an air atmosphere and held at this temperature for 1 h. After the system was purged with argon gas (100 mL/min) for 15 min, a 5% H_2_/Ar gas mixture (100 mL/min) was introduced and the sample was further heated at 250 °C for 45 min. After 2 h at 250 °C, the temperature was then gradually increased to 850 °C at a rate of 2 °C/min for 6 h. According to the Pt–Cu phase diagram, annealing at 850 °C at a composition of approximately PtCu_3_ ensures the formation of a (Pt, Cu) solid solution. The initial annealing in air is to prevent carbon deposit formation on the surface of nanoparticles, while subsequent annealing in a reductive atmosphere prevents oxide formation.

The sample was then cooled to room temperature at a rate of 6 °C/min. Finally, the composite was annealed for 72 h at 500 °C in H_2_/Ar and then rapidly cooled to room temperature, resulting in the final product. Again, the temperature of 500 °C was chosen according to the Pt–Cu phase diagram as it provides appropriate conditions to form an ordered PtCu_3_ alloy, and rapid cooling preserved the crystal structure, obtained during annealing.

### X-ray Diffraction (XRD)

X-ray diffraction patterns were obtained using a PANanalytical X’Pert PRO MPD diffractometer using Cu Kα_1_ radiation (λ = 1.5406 Å). A 2θ range of 10 to 80° was used along with a step size of 0.034° and a holding time of 300 s. The sample was prepared on a Si holder.

### SEM

SEM images were obtained using a SUPRA 35 VP (Carl Zeiss) microscope at 5 kV using detectors for backscattered and secondary electrons. In Figure 1c, the left panel is an SEM image formed with backscattered electrons. The other two panels as well as both images in Figure S1b are mixtures consisting of 56% of the signal coming from backscattered electrons and the rest from secondary electrons. Powder samples were prepared on standard SEM pin mounts (Agar Scientific) covered with conductive carbon tape (Agar Scientific).

### Sample Treatment

A 1 mg/mL suspension was prepared with the powder PtCu_3_/C sample and Milli-Q water. Five microliters of the catalyst suspension was dropcasted on a gold lacey-carbon-coated TEM grid (Agar Scientific). Its treatment consisted of two steps. The first step was acid washing, dipping the grid into 50 mL of 0.1 M perchloric acid (HClO_4_, 70% Rotipuran Supra, Carl Roth, diluted by Milli-Q, 18.2 Ω·cm) for 30 s at room temperature while purging with argon and stirring the electrolyte at 100 rpm. The grid was then washed with Milli-Q water and dried at room temperature.

The second step was carried out in a three-electrode setup with an EmStat4X (PalmSens) potentiostat. For the electrochemical treatment of the sample, deposited on the TEM grid (as above), the modified floating electrode setup was used as the working electrode.^51^ MFE consists of a two-piece Teflon housing, metallic spring, placed between two metallic cones, gas diffusion layer (GDL, 280 μm thickness) with 40% Teflon weight wet proofing (Toray Carbon paper 090, Fuel Cell Store), and a catalyst-coated TEM grid. A reversible hydrogen electrode (HydroFlex) and a Pt mesh were used as reference and counter electrodes, respectively. The experiment was performed in 0.1 M perchloric acid, purged with argon before and during the measurement. After contacting the sample with electrolyte at 0.05 V, the sample was treated by performing 200 cyclic voltammograms between 0.05 and 1.2 V with 300 mV/s. After the experiment, the grid was again washed with Milli-Q water and dried at room temperature.

### STEM and 4D-STEM

STEM images were obtained using a probe Cs-corrected scanning transmission electron microscope Jeol ARM 200 CF. The accelerating voltage was set to 80 kV. For the bright-field and high-angle annular dark-field images, the convergence angle was set to ∼18 mrad, and the collection semiangles were 0–45 and 68–185 mrad, respectively. Energy-dispersive X-ray spectrometry was performed using an SDD Jeol Centurio spectrometer. Electron energy loss spectroscopy maps were acquired with a Dual EELS GIF Quantum Gatan Spectrometer at 200 kV with a beam current of ∼83 pA and a collection semiangle of ∼90 mrad using the Cu L (931 eV) and the Pt M (2122 eV) edges. The raw data was denoised using PCA from Gatan Microscopy Suite. 4D-STEM data sets were acquired as a series of 256 × 256 convergent beam electron diffraction patterns using a Merlin detector (Quantum Detectors, Oxford, U.K.) with a convergence angle of ∼6 mrad. The scan size of the 4D-STEM data was 256 × 256 pixels.

Identical location imaging was performed after synthesis, after acid washing, and after potential cycling activation. Locations of the chosen spots were recorded at different magnifications to aid in finding them during subsequent imaging sessions. All data was recorded under the same conditions.

### 4D-STEM Data Analysis

All data was analyzed using in-house scripts written in Python programming language. Intensities in the recorded diffraction patterns were integrated using virtual apertures in reciprocal space. A circular binary mask covering only the central Bragg disk was used to determine the virtual bright-field image, and an annular binary mask covering other disks was used for the virtual dark-field image.

For subsequent analyses, the raw intensities in the recorded diffraction patterns were preprocessed using a natural logarithm to enhance the lower-intensity features. Pixels with zero values were beforehand replaced by minimum nonzero values, present elsewhere in the data set.

Clustering was used to isolate the diffraction patterns, related to an individual nanoparticle under investigation. Diffraction patterns were clustered using *k*-means clustering, as implemented in the open-source scikit-learn library.^54^ The *k*-means algorithm partitions the diffraction patterns into a user-determined number of clusters, where each pattern can belong to only one cluster, and clusters are determined based on distances between data points in vector space.^55^ A centroid was computed for each cluster, representing the group’s average diffraction pattern. Nanoparticles were segmented using *k*-means clustering with two or three clusters, depending on nanoparticle surroundings in each image. The success of the segmentation was determined by comparing the results to STEM images.

The diffraction patterns, belonging to an individual nanoparticle, were then analyzed using a dimensionality reduction method called non-negative matrix factorization (NMF), as implemented in the scikit-learn library.^54^ NMF decomposes the data by representing it as a product of matrices, one of which includes non-negative eigenvectors of the data.^56^ The eigenvectors can be understood as representative diffraction patterns to describe the commonly present signals within the data set. The number of eigenvectors needs to be specified in advance and should be at least as high as the number of distinct structural features within the data, but not significantly more. It was first estimated by performing PCA and examining the scree plot, which explains the eigenvector variance. Loading maps, obtained with PCA, were normalized to the minimum and maximum value found among all maps. Later, NMF was performed for different numbers of eigenvectors close to the initial estimate, and the final value was chosen by manual evaluation. The number of iterations was set to 1000. The spatial abundance of all eigenvectors was visualized in real space as loading maps that were normalized to the maximum value found among all maps.

### (4D-)STEM Data Simulation

QSTEM software was used to perform simulations of STEM and 4D-STEM data using the multislice method and frozen phonon approximation.^57^ Results were obtained after ten iterations of each simulation. Instrumental parameters matched the experimentally used values and the size of simulated patterns was adjusted to match the size of the experimentally obtained ones.

In simulations for interpreting NMF results, models consisted of disordered-alloy, ordered-alloy, and mixed-phase PtCu_3_ nanoparticles spanning 5.5 nm in diameter. A 10 × 10 grid of diffraction patterns was simulated using a convergence angle of 6 mrad and integrated to yield the average diffraction pattern of each model.

To validate the NMF methodology, the model was a mixed-phase PtCu_3_ nanoparticle spanning 10 nm in diameter. The nanoparticle had a disordered alloy core, representing 50% of the particle volume, and an ordered alloy shell with a Pt-rich surface. A 32 × 32 grid of diffraction patterns was simulated using a convergence angle of 6 mrad and a HAADF-STEM image was simulated using a convergence angle of 24 mrad.

## Data Availability

The code to analyze 4D-STEM data is available at https://github.com/kamsekar/Local-crystal-structure-4DSTEM.

## Supporting Information Available

Additional experimental SEM, STEM, EDX, and 4D-STEM data, simulated 4D-STEM data, additional methodology discussion (PDF)

## Notes

This manuscript was previously submitted to the ChemRxiv preprint server. The citation for the preprint is as follows: A.R.K.; F.R.-Z.; M.B.; A.L.; G.D.; N.H. Identifying Structure–Stability Relationships in Individual Pt-Alloy Nanoparticles Using Identical-Location 4D-STEM and Unsupervised Machine Learning. 2024, chemrxiv-2024-x6f6m. ChemRxiv. 10.26434/chemrxiv-2024-x6f6m (accessed December 19, 2024).
