# Supplementary material for: Structure–Stability Relationships in Pt-Alloy Nanoparticles Using Identical-Location Four-Dimensional Scanning Transmission Electron Microscopy and Unsupervised Machine Learning
Source: ACS Nano. 2025 Jan 7;19(2):2334–44. doi: 10.1021/acsnano.4c12528 (PMC11760175; doi:10.1021/acsnano.4c12528)
Supplement: Supplementary file 1 — nn4c12528_si_001.pdf [file nn4c12528_si_001.pdf]

SUPPORTING INFORMATION

Structure-Stability Relationships in Pt-Alloy Nanoparticles Using Identical-Location Four-Dimensional Scanning Transmission Electron Microscopy and Unsupervised Machine Learning

Ana Rebeka Kamšek[1,2,*], Francisco Ruiz-Zepeda[1], Marjan Bele[1], Anja Logar[1,3], Goran Dražić[1], Nejc Hodnik[1,3*]

[1] Department of Materials Chemistry, National Institute of Chemistry, Hajdrihova 19, 1000 Ljubljana, Slovenia

[2] Faculty of Chemistry and Chemical Technology, University of Ljubljana, Večna pot 113, 1000 Ljubljana, Slovenia

[3] University of Nova Gorica, Vipavska 13, 5000 Nova Gorica, Slovenia

[*] Corresponding authors: ana.rebeka.kamsek@ki.si, nejc.hodnik@ki.si

SI 1. Additional SEM and STEM images of the investigated $PtCu_3$/C sample.

SI 2. Additional (4D-)STEM results of an individual Pt-Cu nanoparticle.

SI 3. Simulated 4D-STEM data, used to explain and validate the NMF results.

SI 4. Comments on using unsupervised algorithms on 4D-STEM data.

SI 5. 4D-STEM investigation of a Pt-Cu nanoparticle in [110] zone axis.

SI 6. Identical-location 4D-STEM after acid washing.

SI 7. Identical-location EDX.

SI 1. Additional SEM and STEM images of the investigated $PtCu_3/C$ sample.

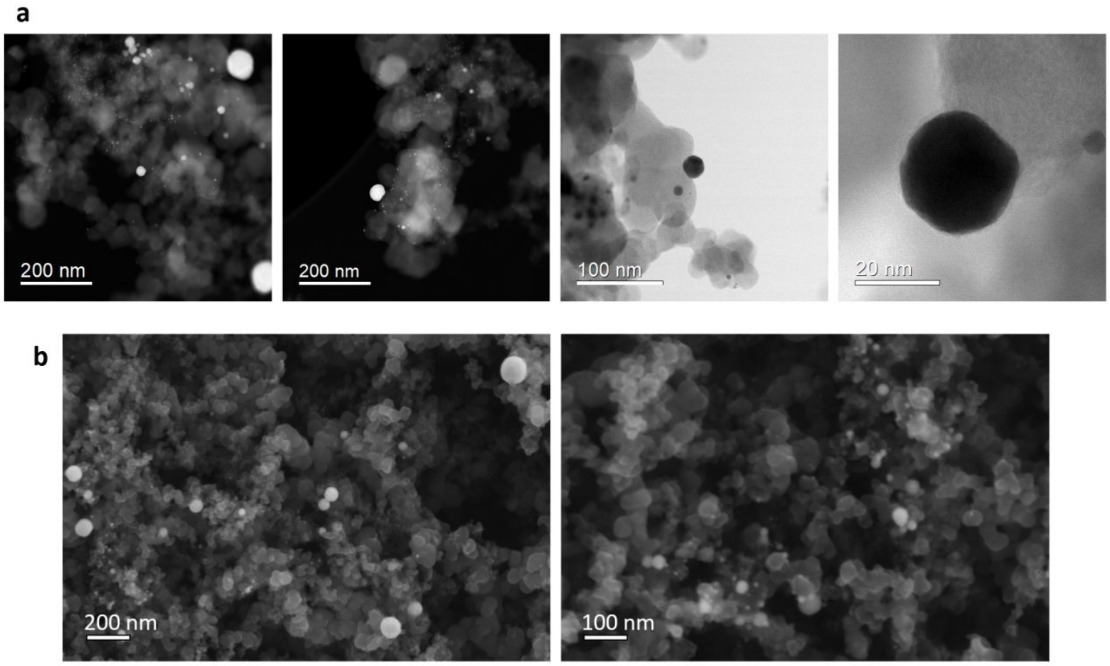

Figure S1. STEM **(a)** and SEM **(b)** images of the $PtCu_3/C$ electrocatalyst.

SI 2. Additional (4D-)STEM results of an individual Pt-Cu nanoparticle.

Figure S2a shows a BF-STEM image of a Pt-Cu nanoparticle in a [111] zone axis. Its average diffraction pattern, computed from the 4D-STEM dataset, is depicted in **Figure S2b** and exhibits a signal with a six-fold symmetry, consistent with the [111] zone axis for a face-centered cubic unit cell.

Virtual imaging was performed to inspect the 4D-STEM data. **Figure S2c** shows a bright-field and a dark-field image, as reconstructed from the 4D-STEM dataset using a circular and an annular virtual detector. Each virtual detector, visualized in **Figure S2d**, was used as a virtual aperture, inside which intensities were integrated to yield a real-space image.

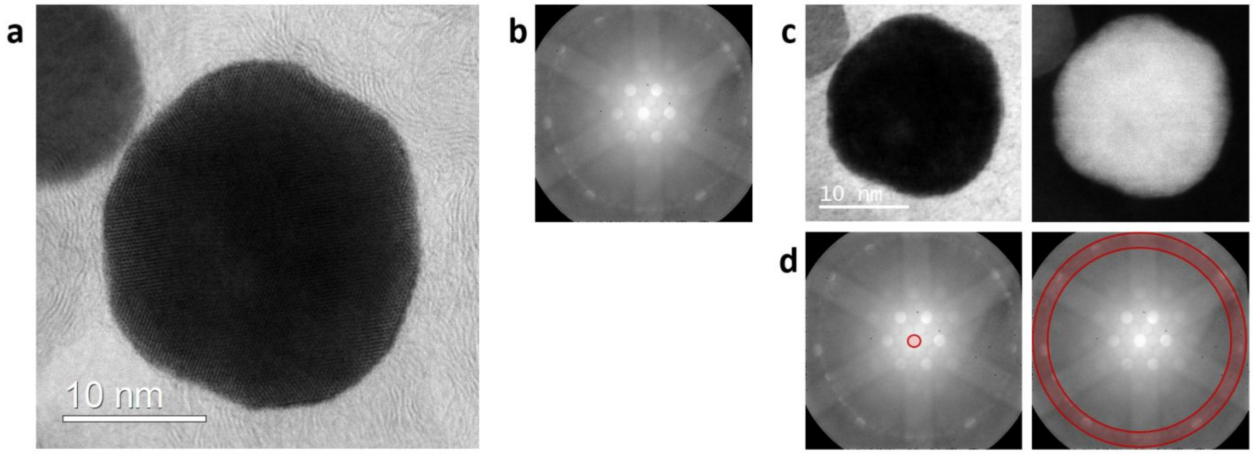

Figure S2. (a) A BF-STEM image of a Pt-Cu nanoparticle. **(b)** Average diffraction pattern, determined with 4D-STEM. **(c)** Reconstructed bright-field (left) and dark-field (right) images from the 4D-STEM data. **(d)** Virtual detectors for reconstructed images in **(c)**, depicted as red masks over the average diffraction pattern.

SI 3. Simulated 4D-STEM data, used to explain and validate the NMF results.

Figure S3 presents three models that were used to obtain simulated diffraction patterns in **Figure 2g**.

In the disordered alloy model in **Fig. S3a**, Pt and Cu atoms are placed randomly. In the ordered alloy model in **Fig. S3b**, their placement is not random, and in the mixed model in **Fig. S3c**, there is a disordered core and an ordered shell, more clearly seen in the cross section matching the [111] zone axis in the bottom row. In the mixed model, the two phases each make up 50 % of the particle volume.

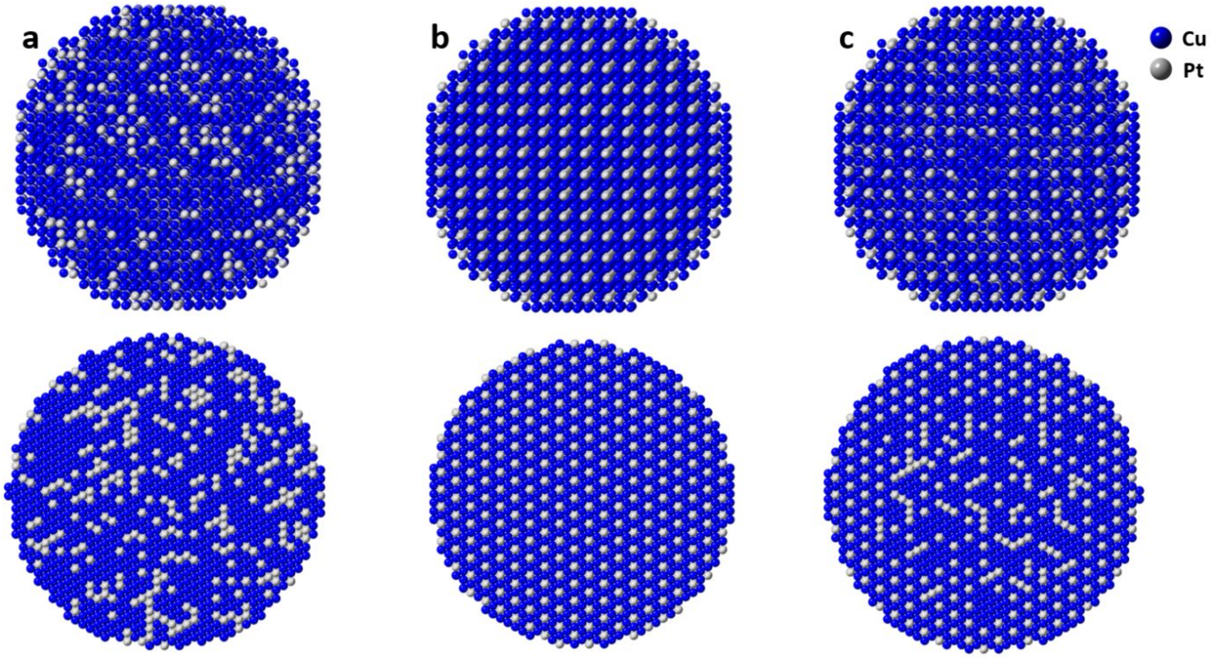

Figure S3. Pt-Cu nanoparticle models close to the [001] zone axis to simulate 4D-STEM datasets (top), and a cross section matching the [111] zone axis (bottom), with **(a)** a disordered Pt-Cu alloy crystal structure, **(b)** an ordered $PtCu_3$ alloy structure, and **(c)** their mixture.

The results of using NMF, a dimensionality reduction algorithm, on experimental 4D-STEM data, were compared to those on a simulated dataset for validation as shown in **Figure S4**. The model was a Pt-Cu nanoparticle spanning approximately 10 nanometers in diameter. The particle had a disordered alloy core, accounting for 50 % of its volume, and an ordered alloy shell. A part of Cu atoms in the outermost few layers of the shell were randomly switched to Pt to obtain a Pt-rich surface.

The NMF results for three components returned patterns, consistent with a non-crystalline signal, an Fm-3m signal, and a signal with highlighted superstructure disks as expected from a Pm-3m structure. Their loading maps predominantly highlighted the vacuum, the Pt-rich surface, and the ordered alloy which is present throughout the image although to different extents, as the ordered shell encapsulates the disordered core in all three dimensions.

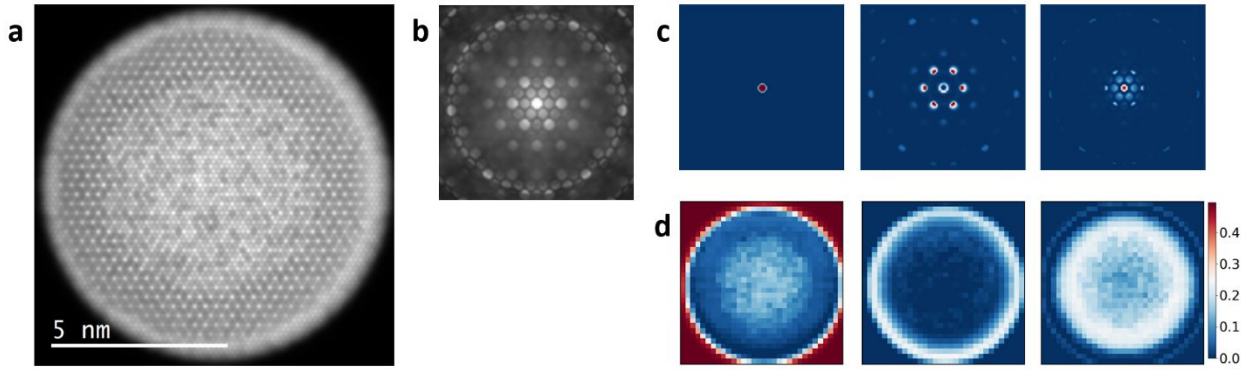

Figure S4. (a) A simulated HAADF-STEM image of a model Pt-Cu nanoparticle. **(b)** Its average diffraction pattern, obtained from simulated 4D-STEM data. **(c, d)** NMF results for the simulated 4D-STEM dataset with calculated diffraction patterns **(c)** and their loading maps with a color scale **(d)**.

SI 4. Comments on using unsupervised algorithms on 4D-STEM data.

Among several considered clustering algorithms, k-means clustering was chosen due to a good compromise between computational complexity and the amount of necessary manual input which consisted only of the number of clusters (and determining the preprocessing). Two or three clusters were sufficient and included a cluster containing the entirety of the investigated nanoparticle while using a larger number of clusters generally resulted in the nanoparticle getting split.

The clustering gave adequate results using the logarithm of the raw data, which enhanced lower-intensity parts of the diffraction patterns and lessened the prominence of the central disk. Taking the square root and masking the central disk were also considered, but did not perform better. In the literature, there does not seem to be a clear consensus on the best data preprocessing which is dependent on the specifics of an individual case, such as instrumental parameters, sample thickness, and crystal structure.

Similarly, the optimal number of clusters depends on the data and should be at least as large as the number of features that should be discerned, but not as large as to risk noise-induced separation. Therefore, this otherwise simple and unsupervised method still requires some manual input.

Among dimensionality reduction methods, NMF was a good choice thanks to its non-negativity constraint since the 4D-STEM detector will also provide us with non-negative counts of electrons. In our case, the minimal data preprocessing, namely taking the natural logarithm of the data, was successful, and other tasks did not yield better results. Similar to clustering, the preprocessing pipeline and the number of eigenvectors needed to be specified in advance.

Despite preprocessing the raw data with a logarithmic function before carrying out the NMF, certain weaker features in the calculated diffraction patterns were difficult to observe. **Figs. 2e** and **4c** therefore include square root values of the calculated patterns, while **Figure S5** contains the NMF results without any post-processing for reference.

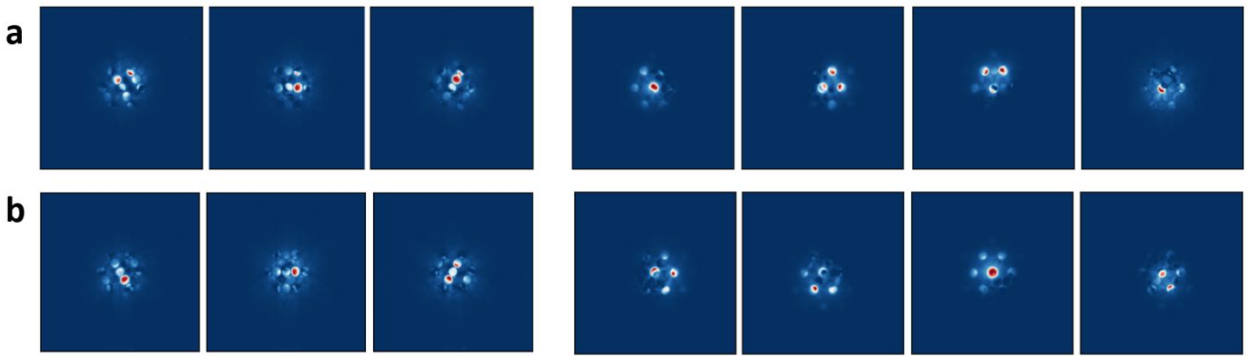

Figure S5. The representative diffraction patterns, determined with NMF, for a Pt-Cu nanoparticle **(a)** after synthesis and **(b)** after potential cycling activation. The intensities were not additionally processed.

The number of NMF eigenvectors was determined for each dataset separately. For the data on one Pt-Cu nanoparticle after synthesis and after sample treatment, as presented in **Figs. 2e** and **4c**, the number of eigenvectors was the same. If the changes to the sample were more drastic, it is possible that it would change to reflect the number of distinct signals in each dataset. A direct correspondence between two sets of results can confirm the reshaping of individual domains within a nanoparticle.

PCA was used to provide a first estimate for the number of components. However, it can also be used as an alternative to NMF in terms of being a dimensionality reduction method that can be used to discern important signals within a dataset. **Figure S6** shows calculated diffraction patterns, determined as principal components, and their corresponding loading maps. While the colormap is still the one, used to visualize NMF results, the loading maps are now normalized to the maximum and minimum values, found amongst all of the maps.

While the calculated diffraction patterns largely follow the logic of highlighting individual Bragg disks, similar to NMF results, they are more difficult to interpret because they include both positive and negative values. Even though PCA can still discern parts of the nanoparticle exhibiting a specific diffraction signal, NMF results can be more easily connected to simulated data and thus explained in terms of crystal structure.

a

b

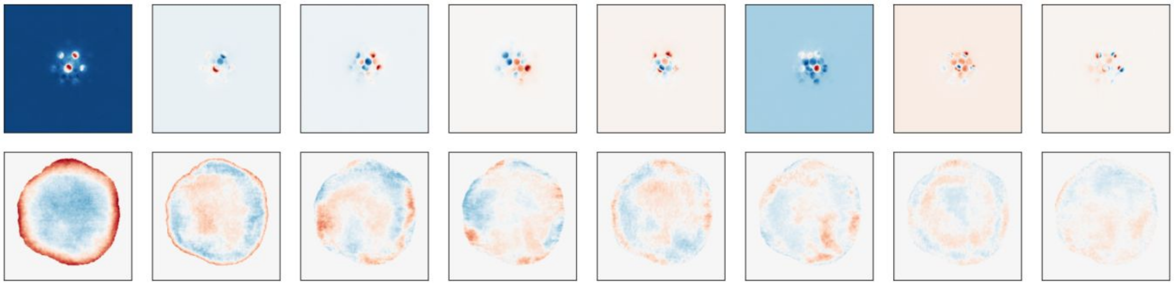

Figure S6. (a) Calculated diffraction patterns, determined as dataset eigenvectors with PCA. **(b)** Loading maps, corresponding to the calculated diffraction patterns. The color scale is normalized to maximum and minimum values in the entire set of loading maps.

SI 5. 4D-STEM investigation of a Pt-Cu nanoparticle in [110] zone axis.

Figure S7 contains the results of a 4D-STEM investigation of a twinned Pt-Cu nanoparticle in a [110] zone axis. In the FFT of the HAADF-STEM image, pairs of intensity maxima signify parallel twinning. K-means clustering was performed (**Figure S7c**), and returned a cluster with Pt-Cu patterns in a [110] zone axis as well as two clusters where a ring signal confirmed the partially amorphous carbon support.

Figure S7e includes the NMF results. Parallel twin boundaries are immediately revealed in loading maps in **Figure S7f**, although they are not easily seen in HAADF-STEM images. The average nanoparticle diffraction pattern in **Figure S7d** already shows that multiple disks overlap and the simulations confirm that telling apart the ordered disks is not as simple, especially in the case of partial ordering. Additionally, parallel twin boundaries increase the number of Bragg disks and subsequently their overlap.

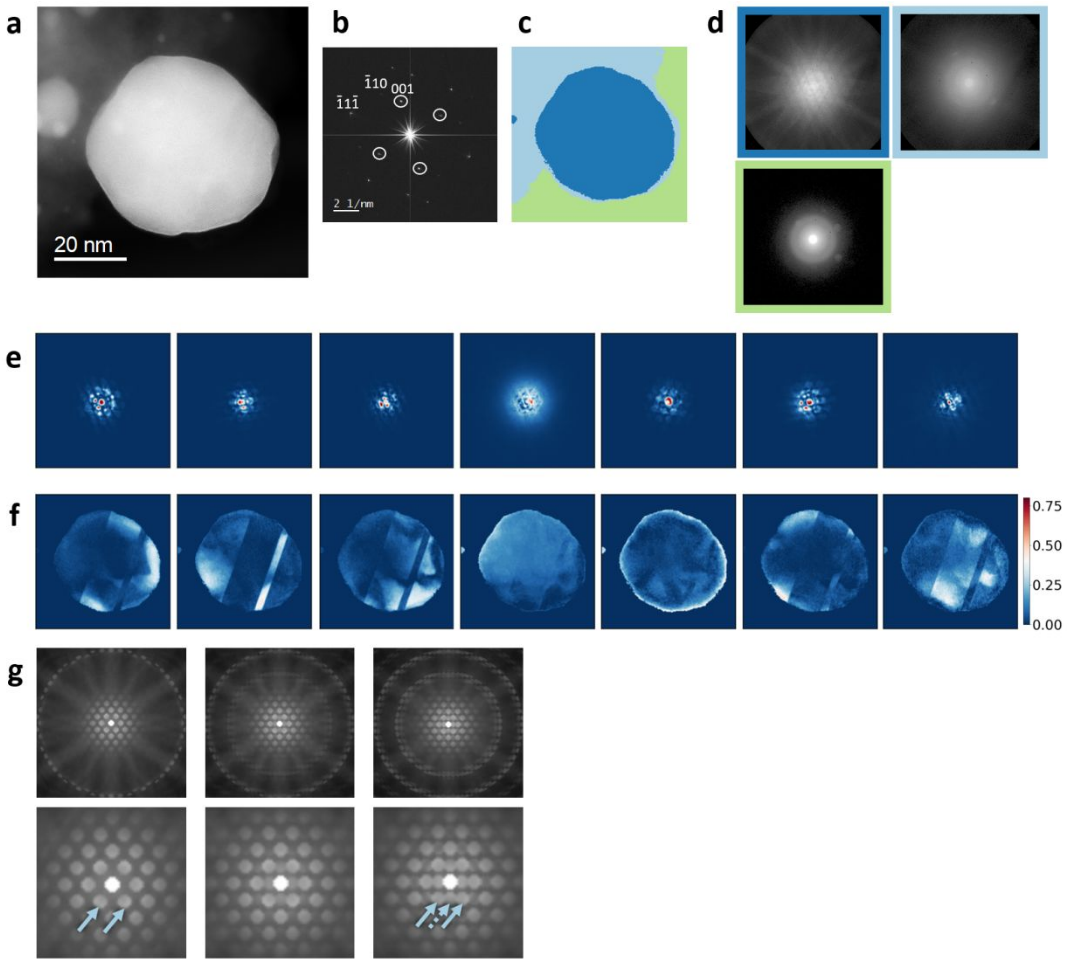

Figure S7. (a) A HAADF-STEM image of a twinned Pt-Cu nanoparticle in the [110] zone axis. **(b)** FFT of the image. Circles denote maxima, belonging to twinned domains. **(c, d)** Clustering with color-coded labels **(c)** and the cluster average diffraction patterns **(d)**. **(e, f)** The representative diffraction patterns, determined with NMF **(e)**, and their loading maps **(f)**. The color scale corresponds to the extent to which each calculated pattern is present in the overall diffraction signal. **(g)** Simulated diffraction patterns of relevant Pt-Cu alloy phases in the [110] zone axis and close-ups of their central areas. From left to right: the disordered alloy (arrows denote characteristic Bragg disks), the 50:50 mixture of the ordered and disordered alloys, and the ordered alloy (dashed arrow denotes a superstructure disk).

SI 6. Identical-location 4D-STEM after acid washing.

An identical location HAADF-STEM image of the studied Pt-Cu nanoparticle after acid washing is shown in **Figure S8a** along with the comparison of particle shape to its as-synthesized shape in **Figure S8b**. The particle silhouettes were aligned manually. Minor shrinkage and reshaping can be observed.

Like previously, k-means clustering (**Figure S8c**) successfully segmented 4D-STEM data into a group of patterns, belonging to the Pt-Cu nanoparticle, and a group with the rest of the patterns. The nanoparticle average diffraction pattern exhibits the same six-fold symmetry but appears to be slightly rotated compared to the result after synthesis.

Figure S8e includes the NMF results from the nanoparticle from the second dataset, namely the calculated diffraction patterns and corresponding loading maps in **Figure S8f**. The calculated diffraction patterns follow the same logic as those after synthesis in **Figure 2e**, and the ordered alloy signal can be recognized in some of them. There are small discrepancies when comparing the two sets of calculated patterns, most likely due to a slight rotation of the nanoparticle.

When comparing the loading maps in **Figure S8f** to the ones from the first dataset in **Figure 2f**, it is evident that several of them directly correspond to each other. Specifically, the ordered alloy loading maps show domains of similar shapes, although no major changes can be immediately noticeable.

Based on the EDX investigation, presented in **Figure S9**, even our mild acid-washing protocol caused a slight enrichment of the surface with Pt. However, this is difficult to observe in 4D-STEM results, as the difference between a Pt-Cu disordered alloy signal and a signal with the same symmetry but a slightly different chemical composition can be very subtle. Therefore, little additional chemical information is available to compare the state before and after acid washing.

The calculated diffraction patterns from the first dataset in **Figure 2e** can in principle also be used for creating loading maps from the second one. That way, the same crystal structures can be searched for and changes quantified. However, when the experimental data exhibits systematic changes, like a uniform rotation, sample drift, or changes in imaging conditions, the loading maps cannot be expected to provide meaningful information.

Figure S8g shows loading maps for the second dataset, determined for calculated diffraction patterns from the first dataset. While some particle domains can still be discerned from the maps, it is obvious that the changes are large enough to prevent us from searching for the same signals in different datasets.

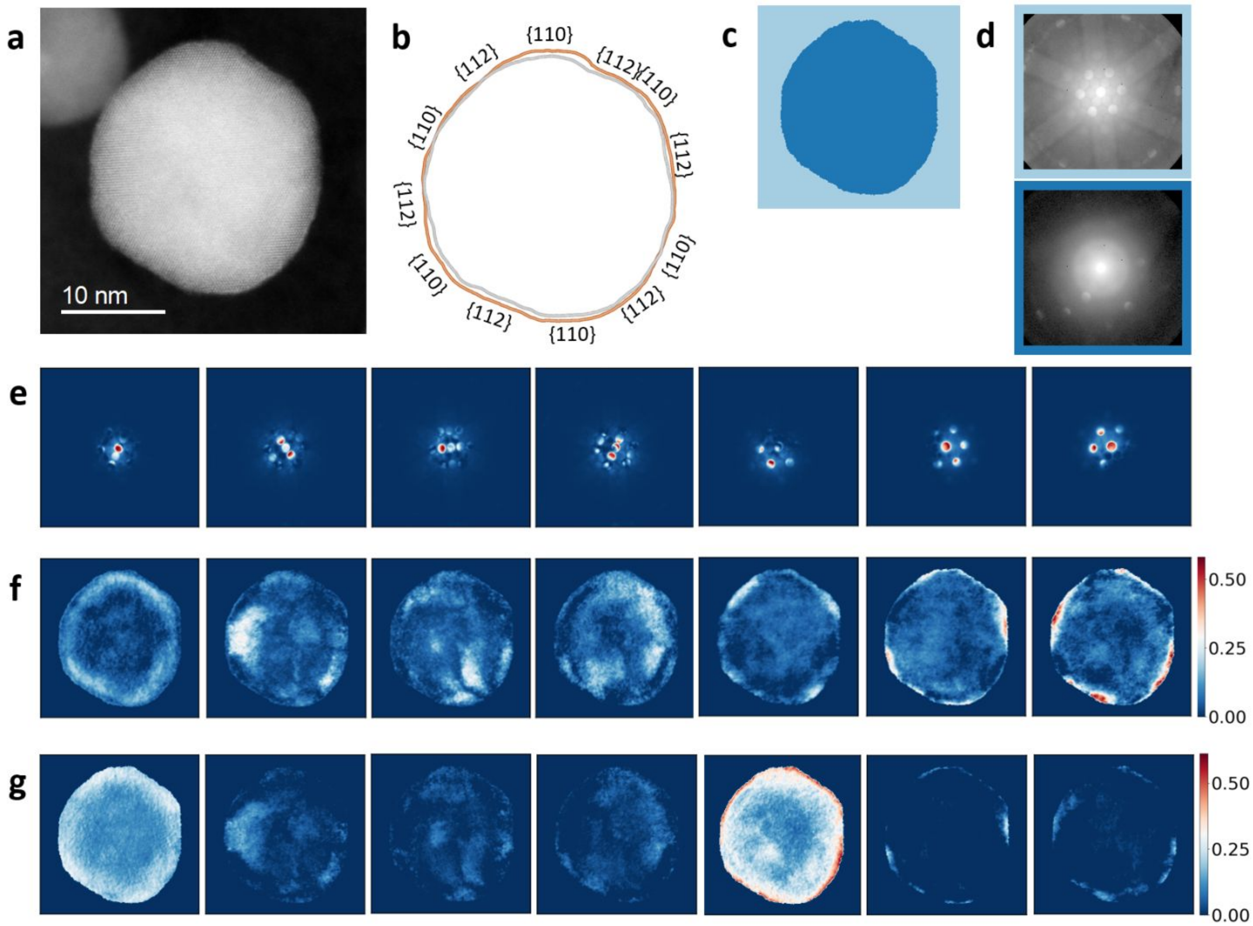

Figure S8. (a) HAADF-STEM image of a Pt-Cu nanoparticle after acid washing. **(b)** Overlaid silhouettes of the nanoparticle before (red) and after (gray) acid washing with Miller indices of crystal plane families. **(c, d)** Clustering with color-coded labels **(c)** and the cluster average diffraction patterns with a border of the same color as the corresponding label **(d)**. **(e, f)** The representative diffraction patterns, determined with NMF **(e)**, and their loading maps **(f)**. The color scale corresponds to the extent to which each calculated pattern is present in the overall diffraction signal. **(g)** Loading maps from the dataset after acid washing, as determined for calculated diffraction patterns before acid washing.

SI 7. Identical-location EDX.

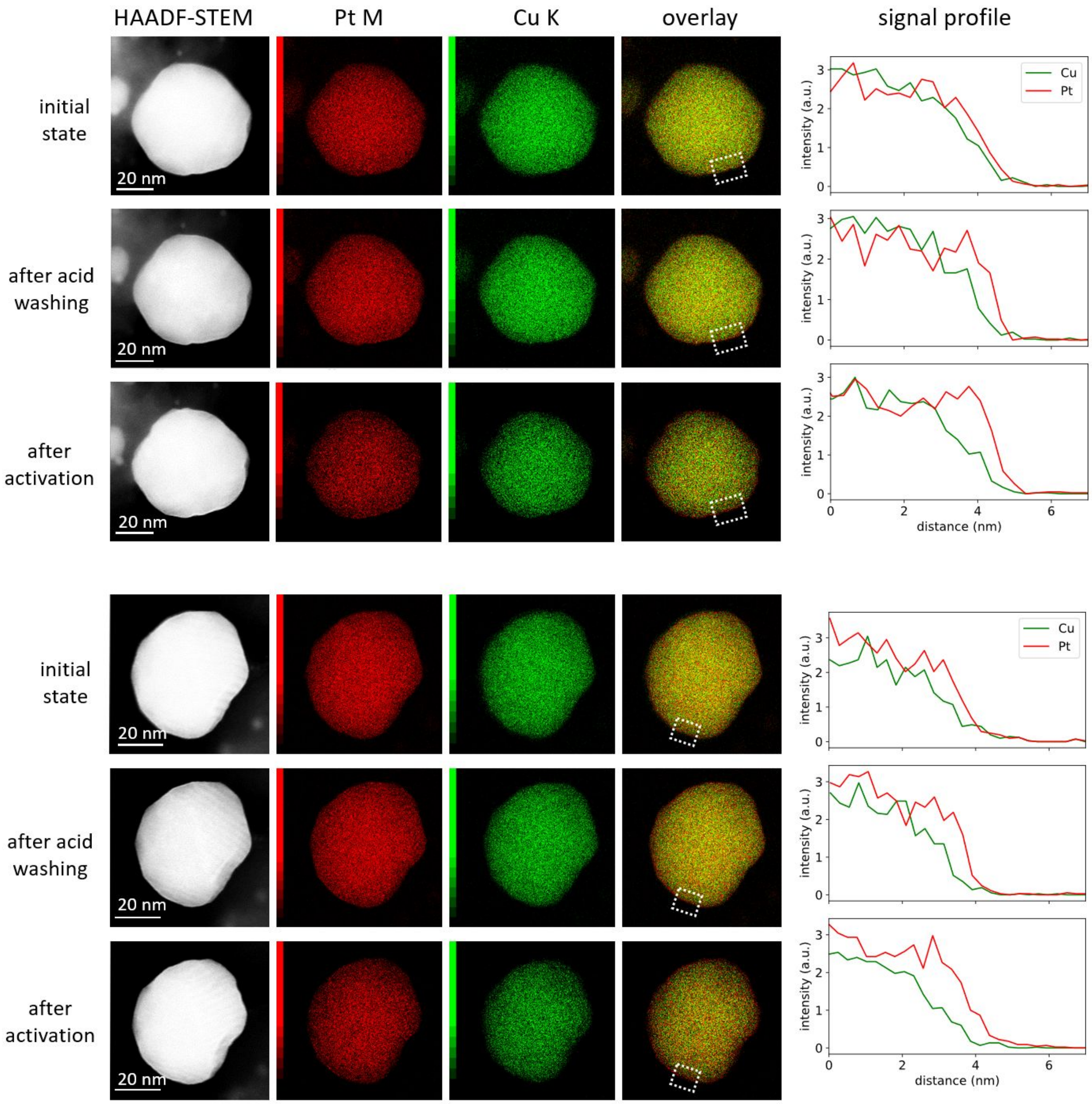

Figure S9. Identical-location EDX results for two Pt-Cu nanoparticles. The Pt signal is shown in red, Cu in green, and the overlay panels contain both signals. For the regions, denoted with white dashed rectangles in the overlay panels, Pt and Cu profiles are plotted to show a progressively more prominent Pt signal with each sample treatment step signifying the formation of a Pt-rich surface.
